# Supplementary material for: Involvement of G-Protein-Coupled Receptor 40 in the Inhibitory Effects of Docosahexaenoic Acid on SREBP1-Mediated Lipogenic Enzyme Expression in Primary Hepatocytes
Source: Int J Mol Sci. 2019 May 28;20(11):2625. doi: 10.3390/ijms20112625 (PMC6600346; doi:10.3390/ijms20112625)

Supplementary Figure 1

A

GO term	Benjamini
Lipid metabolic process	5.5E-42
Cholesterol biosynthetic process	6.5E-21
Oxidation-reduction process	7.6E-21
Sterol biosynthetic process	1.6E-20
Fatty acid metabolic process	3.3E-20
Steroid metabolic process	1.1E-18
Steroid biosynthetic process	5.4E-18
Fatty acid biosynthetic process	3.8E-15
Cholesterol metabolic process	5.9E-14
Metabolic process	6.4E-11

GO term	Benjamini
Lipid metabolic process	5.2E-14
Fatty acid metabolic process	6.5E-14
Fatty acid metabolic process	2.7E-09
Fatty acid biosynthetic process	2.6E-07
Acetyl-CoA metabolic process	4.3E-06

B

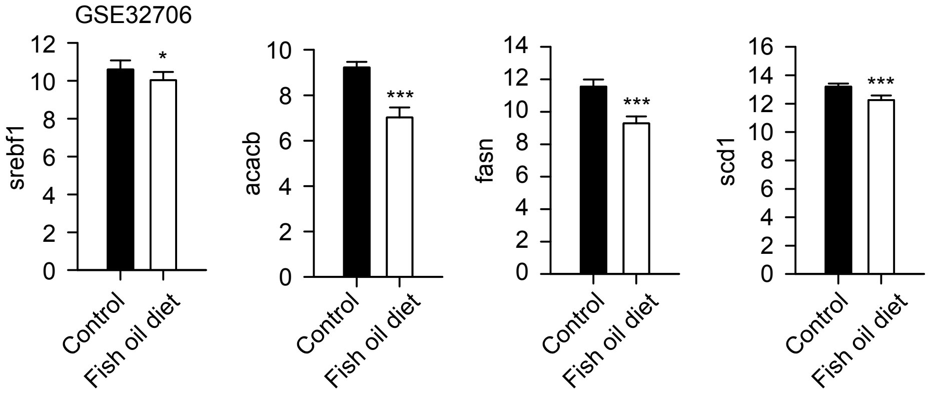

Supplementary Figure 2

A

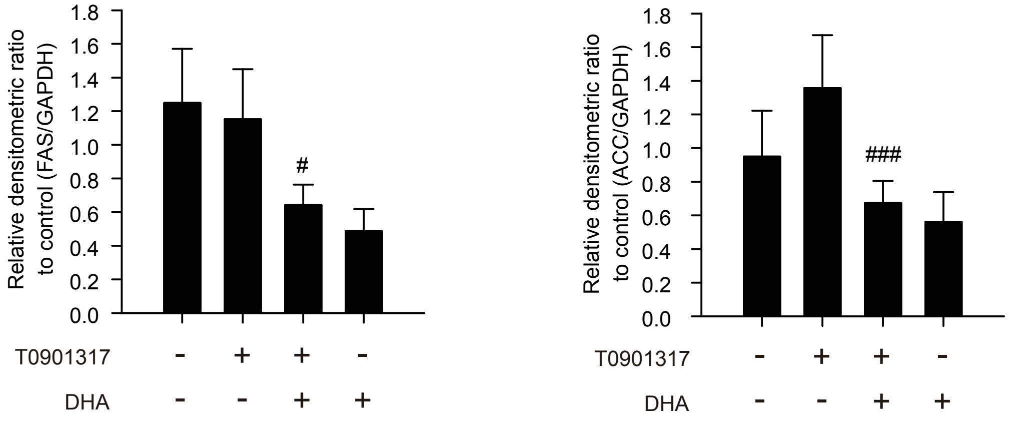

B

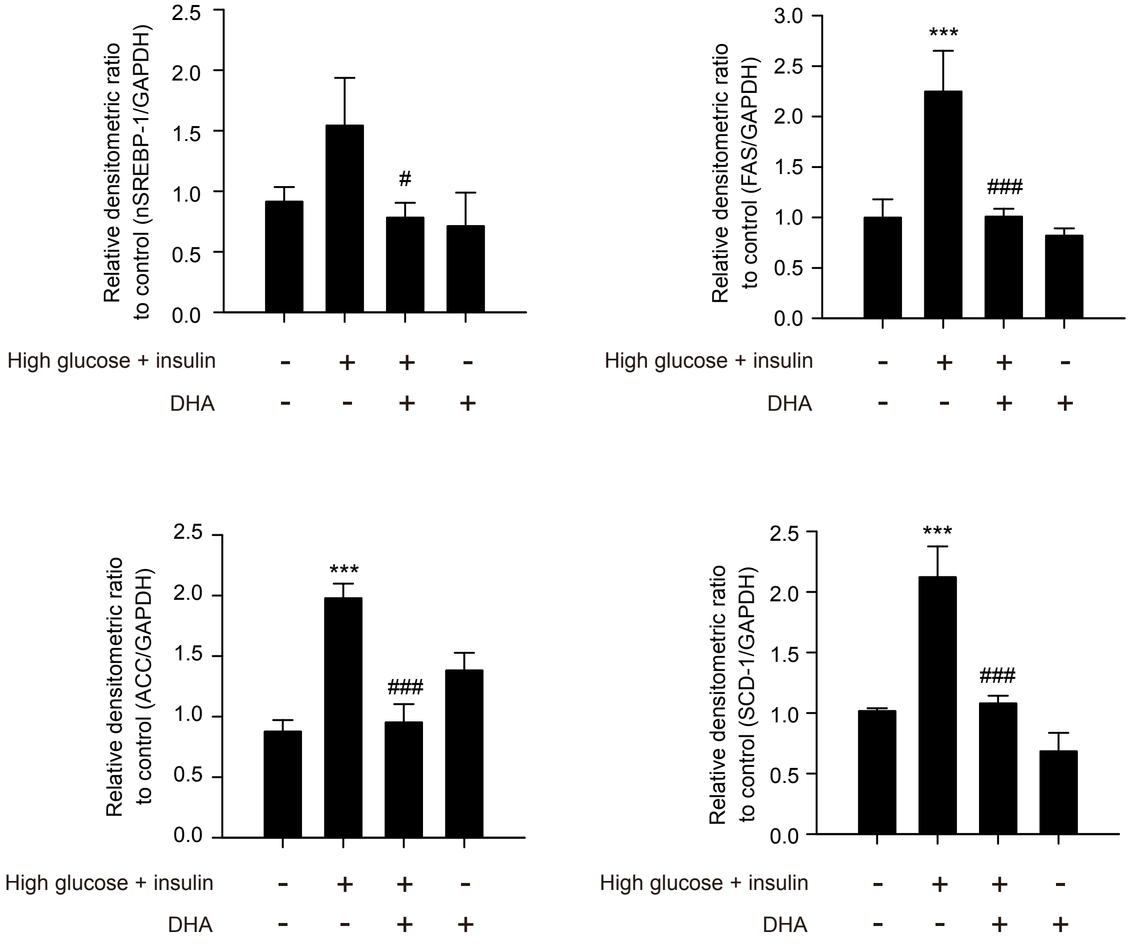

Supplementary Figure 3

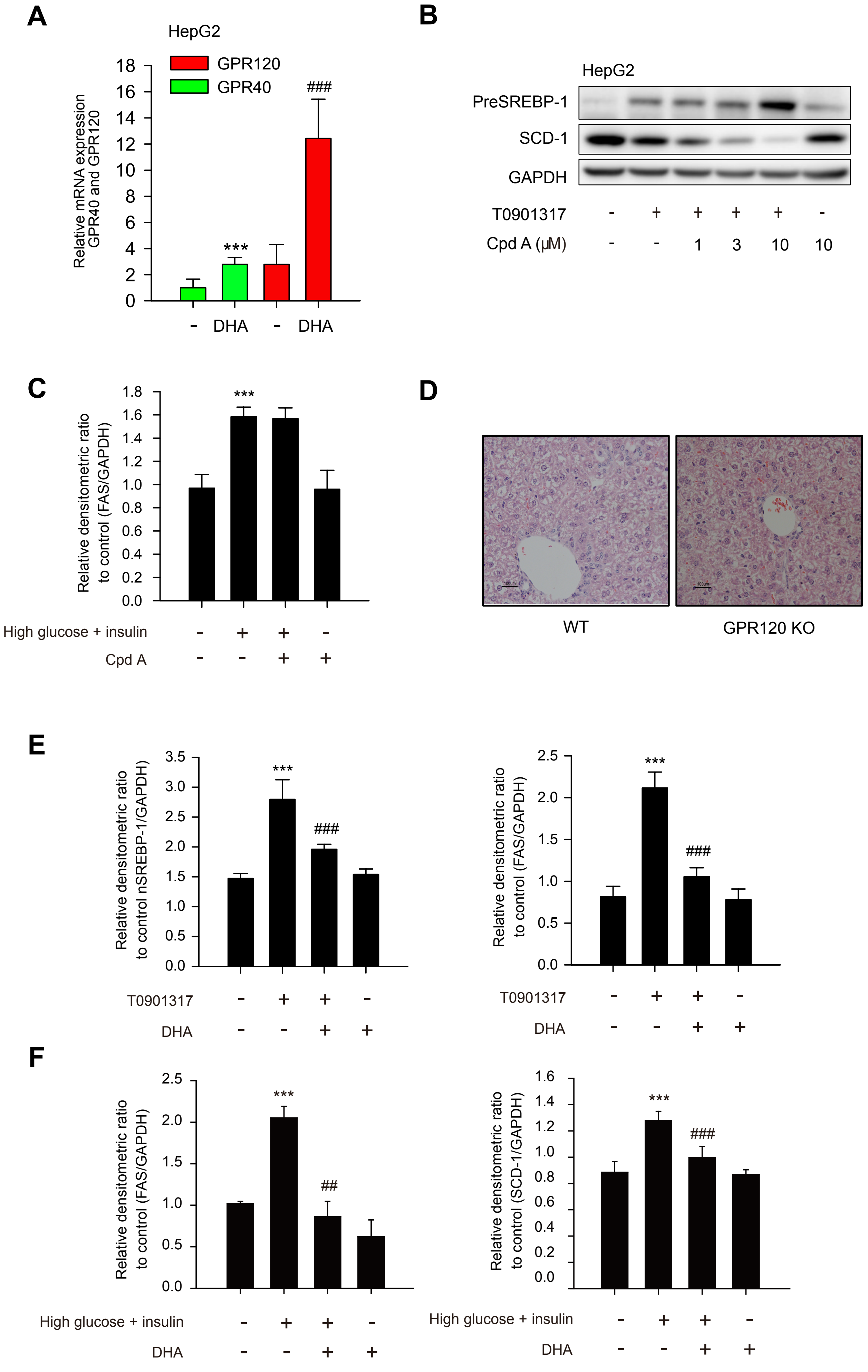

Supplement: Supplementary file 1 [file ijms-20-02625-s001.pdf]
